# The effect of weight change on death and cardiovascular events after Roux-en-Y gastric bypass

**DOI:** 10.1093/bjs/znaf170

**Published:** 2025-08-09

**Authors:** Erik Stenberg, Erik Näslund, Yang Cao, Johan Ottosson, Ingmar Näslund

**Affiliations:** Department of Surgery, Faculty of Medicine and Health, Örebro University, Örebro, Sweden; Division of Surgery, Department of Clinical Sciences, Danderyd Hospital, Karolinska Institute, Stockholm, Sweden; Clinical Epidemiology and Biostatistics, School of Medical Sciences, Örebro University, Örebro, Sweden; Unit of Integrative Epidemiology, Institute of Environmental Medicine, Karolinska Institute, Stockholm, Sweden; Department of Surgery, Faculty of Medicine and Health, Örebro University, Örebro, Sweden; Department of Surgery, Faculty of Medicine and Health, Örebro University, Örebro, Sweden

## Abstract

**Background:**

Weight changes after Roux-en-Y gastric bypass (RYGB) follow different trajectories, but the effects of different trajectories on death and cardiovascular events are largely unknown. The aim of the current study was therefore to evaluate the effects of weight changes after RYGB on cardiovascular events and mortality rate.

**Methods:**

This cohort study included patients who underwent primary RYGB in Sweden from 2007 to 2018 with a complete registration of weight at baseline, at nadir weight loss and 5-year follow-up (*n* = 25 230) with a mean BMI of 42.1 ± 5.2 kg/m^2^, age 42.5 ± 11.2 years, and 19 420 (77%) women. Patients were stratified based on weight change from nadir weight loss. The main outcome measures were major cardiovascular event (MACE) or death.

**Results:**

Over a mean follow-up of 10.6 years, 1276 patients experienced at least one episode of a MACE, and 707 died. An increased risk for death and MACE was seen in patients with continued weight loss after nadir (adjusted HR compared to recurrent weight gain of 0–20% of weight lost at nadir among patients who initially lost 20–35% total weight (TWL): 1.80 (1.41–2.31) and 1.62 (1.35–1.94) respectively), and for patients who experienced >50% recurrent weight gain from nadir (adjusted HR compared to patients with recurrent weight gained 0–20% TWL: 1.61 (1.07–2.43) and 1.48 (1.09–2.00) respectively).

**Conclusion:**

Continued weight loss and significant recurrent weight gain after the initial weight nadir were both associated with a higher risk for MACE and death after RYGB. These should be considered non-desirable weight trajectories requiring further clinical evaluation and increased support.

## Teaser

In this nationwide cohort of patients who underwent Roux-en-Y gastric bypass, patients experiencing more than 50% recurrent weight gain and those who continue to lose weight after the initial weight-loss phase had higher risk for cardiovascular events and death compared to those who regained 0–50% of their lost weight. Significant recurrent weight gain but also continued weight loss should be considered non-desirable trajectories requiring clinical evaluation and increased support.

## Introduction

Obesity is associated with reduced life expectancy and an increased risk of cardiovascular events^1^. Metabolic bariatric surgery (MBS) may reduce the risk for cardiovascular events and improve life expectancy^2–4^. After Roux-en-Y gastric bypass surgery (RYGB), patients tend to lose weight rapidly and reach a plateau of weight nadir somewhere between 1 and 2 years after the operation. Although some recurrent weight gain is common after reaching a nadir weight loss, most patients retain a good and sustainable weight loss long term^5^.

However, some patients experience a more prominent recurrent weight gain, which may result in suboptimal clinical response and recurrence of obesity-related complications^6^. A recurrent weight gain exceeding 20% of the maximum weight loss has been associated with increased progression of type-2 diabetes (T2D) and reduced quality of life (QOL)^7^. Although most patients follow an expected weight trajectory including minor recurrent weight gain after reaching nadir weight loss, a small proportion of patients continue to lose weight beyond 1–2 years after surgery^5^. The effect of these different trajectories on mortality rate and major cardiovascular events (MACE) is largely unknown.

The aim of this registry study was to evaluate the association between recurrent weight gain and continued weight loss after weight loss nadir on mortality, and major cardiovascular events after RYGB.

## Methods

This is a cohort study including patients who underwent primary RYGB in Sweden from January 2007 until March 2018 identified through the Scandinavian Obesity Surgery Registry (SOReg). SOReg is a continuously validated national research and quality registry covering virtually all MBS performed in Sweden with high-quality of data^8^. By using personal identification numbers unique to all Swedish residents, SOReg was linked to the National Patient Registry (for hospital admissions and outpatient contact in specialized care)^9^, the National Prescribed drugs registry (covering all dispensed prescribed drugs in Sweden)^10^, the longitudinal integrated database for health insurance, labour market studies (to cover socioeconomic data at an individual level)^11^, and the total population registry (to cover death and migration)^12^.

Patients who migrated or died during 5 years after surgery, patients without registered weight at nadir and 5 years after surgery, or who were pregnant at the time of follow-up were excluded.

### Surgical technique

The surgical technique for laparoscopic RYGB was highly standardized in Sweden during the study period, using an antecolic, antegastric technique as described previously^13^. Preoperative intentional weight reduction during the immediate weeks before surgery is also widely accepted and used as a routine following current guidelines from the Enhanced Recovery After Surgery Society^14^.

### Definition of variables

Age, sex, and BMI were based on data from the SOReg. Baseline BMI was defined as BMI before the start of the preoperative weight-reducing diet in the immediate weeks before surgery. T2D, hypertension, sleep apnoea, dyslipidaemia, and depression were based on data from the SOReg defining these co-morbidities as a diagnosed disease with pharmacological treatment or nocturnal continuous positive airway pressure treatment. Cardiovascular co-morbidity was based on data from the national patient registry defined as a previous diagnosis of ischaemic heart disease, heart failure, or arrhythmic heart disease. Smoking history was based on data from the SOReg and categorized as no smoking or a history of smoking.

Disposable income (total taxable income minus taxes and negative transfers) was based on data from Statistics Sweden, adjusted for the 2023 consumer price index, and divided into quartiles based on all patients operated with bariatric surgery from 2007. The highest level of education was divided into three categories based on the highest level of education completed at the time of the intervention: primary education (≥9 years of schooling), secondary education (10–12 years of schooling), or higher education.

Nadir weight loss was defined as the greatest weight loss at 1–2 years after surgery. Weight difference from nadir was defined by the percentage increase in weight from nadir weight loss (in percentage of the weight lost) and defined as continued weight loss (<0% weight change), 0–20% recurrent weight gain, 20–50% recurrent weight gain, and >50% recurrent weight gain based on a previous study suggesting 20% recurrence of the maximum weight-loss as the best performing definition of recurrent weight gain^7,15^.

### Outcomes

The main outcome was all-cause death based on data from the total population registry. The secondary outcome was the occurrence of MACE, defined as hospitalization for acute coronary syndrome, cerebrovascular events, or all-cause death.

### Statistics

Continuous variables are presented as means(s.d.). Categorical variables are presented as numbers and proportions (%). The incidence of primary and secondary outcomes was evaluated using incidence rates with risk estimated through a multivariable Cox regression model. The proportional hazards assumption was evaluated using Schoenfeld residuals and tested with the Grambsch–Therneau method^16^. Additionally, residuals were visually inspected over time. No statistically significant violations of the proportional hazards assumption were detected. The model was stratified by nadir total weight loss and adjusted for age, sex, baseline BMI, co-morbid disease, disposable income, level of education, and smoking. Hazard ratios and their 95% confidence intervals were reported to quantify relative risks and associated uncertainties. Patients were followed until emigration, death, or 31 December 2022, whichever occurred first. Missing values for income, education, and smoking were grouped under a separate category ‘Unknown’ and included in the regression analysis^17^. Sensitivity analyses were conducted to assess the robustness of the results by comparing results obtained from the complete case analysis and imputed data sets. Missing data were addressed using multiple imputations by chained equation. In this study, we performed five imputations to generate multiple imputed data sets, ensuring robust estimation of missing values. After imputation, pooled estimates were derived using Rubin’s rule, which combines results from multiple imputed data sets to produce valid statistical interference^18^. Further sensitivity analyses were performed after the exclusion of all patients who were diagnosed with cancer before the 5-year follow-up and extended to one year after this follow-up to account for patients with symptoms of cancer who were not yet diagnosed.

SPSS version 29 (IBM, Armonk, NY, USA), Stata version 17.0 (StataCorp, College Station, TX, USA), and R version 4.4.1 (R Foundation for Statistical Computing, Vienna, Austria) were used for statistical analyses.

## Results

From January 2007 until March 2018, 53,339 patients underwent a primary RYGB. After the exclusion of 699 patients who died within 5 years after surgery, 383 patients who underwent a conversion procedure at any time, 277 who were pregnant at follow-up registration, and 26 750 with missing registration of either nadir weight loss or at 5 years after surgery; 25 230 patients remained in the study group (*Table 1*).

**Table 1:** Baseline characteristics of the study group

*N*	25230
Age, years, mean(s.d.)	42.5(11.19)
**Sex, *n* (%)**	
Men	5810 (23.0%)
Women	19 420 (77.0%)
BMI, kg/m^2^, mean(s.d.)	42.1(5.19)
**Co-morbidity**	
Cardiovascular co-morbidity, *n* (%)	1266 (5.0%)
Sleep apnoea, *n* (%)	2677 (10.6%)
Hypertension, *n* (%)	7134 (28.3%)
Type-2 diabetes, *n* (%)	3887 (15.4%)
Dyslipidaemia, *n* (%)	2766 (11.0%)
Depression, *n* (%)	3471 (13.8%)
**Smoking status, *n* (%)**	
Yes	6650 (26.4%)
No	16 906 (67.0%)
Unknown	1674 (6.6%)
**Level of education, *n* (%)**	
Low	4040 (16.0%)
Mid	15 359 (60.9%)
High	5720 (22.7%)
Unknown	111 (0.4%)
**Income, *n* (%)**	
Q1	6549 (26.0%)
Q2	6333 (25.1%)
Q3	6936 (27.5%)
Q4	5405 (21.4%)
Unknown	7 (0.0%)

*N* = number; Q = quartile.

**Fig. 1:**
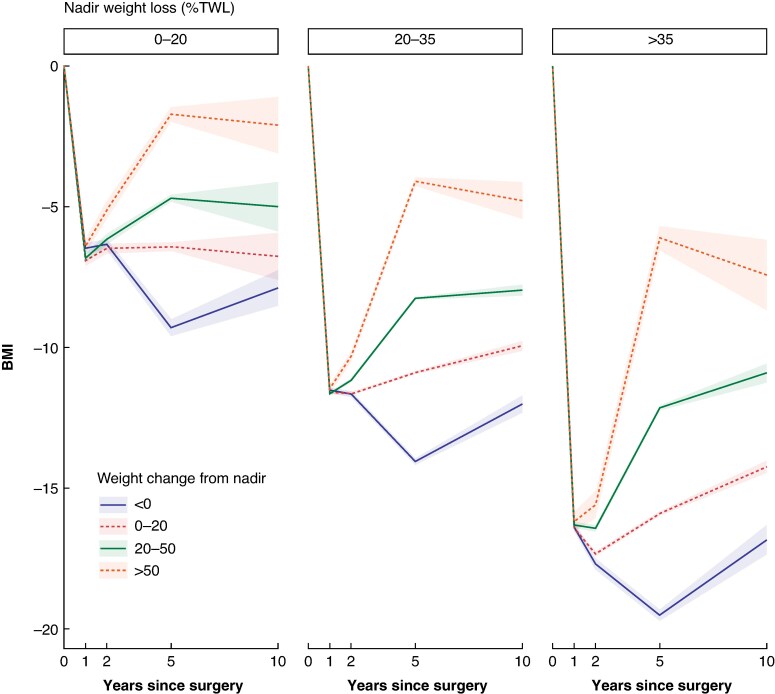
**BMI loss trajectories up to 10 years after surgery, depending on weight change from the expected nadir, stratified by total weight loss at nadir**. Ribbons represent 95% confidence intervals

The mean maximum postoperative total weight loss was 33.4 ± 8.04% declining to 28.5 ± 9.68% at 5 years after surgery. At 5 years after surgery, 4377 patients (17.3%) had continued to lose weight after the expected nadir, whereas 11 704 patients (46.4%) had a recurrent weight gain of 0–20% from their maximum weight loss, 7791 (30.9%) had a recurrent weight gain of 20–50%, and 1358 (5.4%) had regained more than 50% from their maximal weight loss (*Fig. 1*). During a mean follow-up time of 10.6 ± 2.44 years, 1276 patients experienced a MACE, and 707 died.

Patients who continued to lose weight after the expected nadir had slightly higher baseline BMI (*P* < 0.001), a higher prevalence of metabolic co-morbidities and depression (*P* < 0.001), a higher rate of smoking (*P* < 0.001), and slightly lower education (*P* < 0.001) and income (*P* < 0.001) at baseline compared to those with 0–50% recurrent weight gain. Patients with >50% recurrent weight gain were younger (*P* < 0.001), with slightly higher BMI (*P* = 0.002), more often had depression (*P* < 0.001), and slightly lower income (*P* < 0.001) at baseline compared to those with 0–50% recurrent weight gain (*Tables S1*).

### Mortality rate

The incidence rate for mortality in the study group was 2.64/1000 person-year (95% c.i. 2.46 to 2.85). A higher risk was seen among patients who lost a smaller proportion of their weight at the nadir. Continued weight loss after the expected nadir was associated with a higher risk for death in patients who lost >20% of their total weight at the nadir, whereas no significant difference was seen for patients who lost <20% of their total weight at the nadir. For patients with recurrent weight gain, an increased risk of mortality was only seen among patients who lost 20–35% of their total weight at nadir and thereafter experienced >50% recurrent weight gain (*Table 2*). The most common cause of death was cardiovascular events (*n* = 208, 29.4% of all deaths), followed by cancer (*n* = 175, 24.8%) and suicide, intoxication, or accidents (*n* = 144, 20.4%), without any major difference between the groups (*Tables S2–S3*).

**Table 2:** Incidence rate and risk for death based on nadir total weight loss and weight change from nadir

Weight change from nadir	Incidence rate/1000 person-years	HR (95% c.i.)	Adjusted HR (95% c.i.)	*P* *
**Initial total weight loss <20%**				
<0%	6.91 (4.77–10.01)	1.49 (0.80–2.75)	1.48 (0.79–2.76)	0.222
0–20%	4.69 (2.87–7.66)	Ref	Ref	Ref
20–50%	4.32 (2.64–7.05)	0.89 (0.45–1.79)	0.78 (0.38–1.59)	0.496
>50%	3.53 (1.76–7.05)	0.73 (0.31–1.70)	0.87 (0.36–2.07)	0.745
**Initial total weight loss 20–35%**				
<0%	4.34 (3.64–5.18)	1.74 (1.37–2.23)	1.80 (1.41–2.31)	<0.001
0–20%	2.42 (2.05–2.87)	Ref	Ref	Ref
20–50%	2.39 (1.98–2.87)	1.00 (0.78–1.28)	1.02 (0.80–1.32)	0.852
>50%	3.03 (2.09–4.39)	1.27 (0.85–1.91)	1.61 (1.07–2.43)	0.022
**Initial total weight loss >35%**				
<0%	3.73 (2.86–4.86)	2.17 (1.57–3.00)	1.93 (1.39–2.68)	<0.001
0–20%	1.66 (1.38–2.01)	Ref	Ref	Ref
20–50%	2.26 (1.79–2.85)	1.38 (1.02–1.87)	1.28 (0.95–1.74)	0.105
>50%	2.40 (1.14–5.02)	1.48 (0.69–3.18)	1.42 (0.66–3.06)	0.367

^*^Adjusted for age, sex, preoperative BMI, cardiovascular disease, sleep apnoea, hypertension, diabetes, dyslipidaemia, depression, income, education, and smoking.

### Major adverse cardiovascular events

The incidence rate for MACE in the study group was 4.83/1000 person-year (95% c.i. 4.57 to 5.10). A higher risk was seen among patients who lost a smaller proportion of their weight at the nadir. Continuous weight loss after the expected nadir was associated with higher risk of death in patients who lost >20% of their total weight at the nadir, whereas no significant difference was seen for patients who lost <20% of their total weight at the nadir. For patients with recurrent weight gain, an increased risk for mortality was only seen among patients who lost 20–35% of their total weight at the nadir and thereafter experienced >50% recurrent weight gain (*Table 3*).

**Table 3:** Incidence rate and risk for major cardiovascular adverse events based on nadir total weight loss and weight change from nadir

Weight change from nadir	Incidence rate/1000 person-years	HR (95% c.i.)	Adjusted HR (95% c.i.)	*P* *
**Initial total weight loss <20%**				
<0%	10.09 (7.40–13.76)	0.99 (0.63–1.56)	1.05 (0.66–1.67)	0.835
0–20%	10.21 (7.30–14.29)	Ref	Ref	
20–50%	9.16 (6.51–12.89)	0.88 (0.55–1.42)	0.84 (0.52–1.37)	0.489
>50%	6.24 (3.70–10.54)	0.60 (0.32–1.12)	0.73 (0.39–1.38)	0.333
**Initial total weight loss 20–35%**				
<0%	7.70 (6.73–8.80)	1.53 (1.28–1.83)	1.62 (1.35–1.94)	<0.001
0–20%	4.96 (4.41–5.59)	Ref	Ref	Ref
20–50%	4.64 (4.06–5.30)	0.94 (0.79–1.12)	0.97 (0.81–1.16)	0.728
>50%	5.48 (4.15–7.22)	1.11 (0.82–1.50)	1.48 (1.09–2.00)	0.012
**Initial total weight loss >35%**				
<0%	5.48 (4.40–6.82)	1.81 (1.40–2.35)	1.52 (1.16–1.98)	0.002
0–20%	2.98 (2.58–3.43)	Ref	Ref	Ref
20–50%	3.89 (3.26–4.65)	1.32 (1.06–1.66)	1.21 (0.96–1.52)	0.103
>50%	3.43 (1.85–6.39)	1.18 (0.62–2.22)	1.10 (0.58–2.07)	0.744

^*^Adjusted for age, sex, preoperative BMI, cardiovascular disease, sleep apnoea, hypertension, diabetes, dyslipidaemia, depression, income, education and smoking.

### Sensitivity analyses

After multiple imputations of missing data, the cohort had a lower age, and slightly lower incidence of co-morbidities but no differences in socioeconomic status compared to the study group (*Table S4*). The associations between weight changes and risk for mortality and MACE remained in these analyses (*Tables S5–S7*). Excluding patients who were diagnosed with cancer during the first 6 years after surgery from the study group did not alter the associations (*Table S8–S9*).

## Discussion

Patients who lost more weight at nadir had lower incidences of MACEs and reduced risk for mortality in concurrence with previous reports^19^. Recurrent weight gain was associated with increased risk for MACE and death. Patients with greater nadir weight loss also had an increased risk for MACE and death with continued weight loss after the expected nadir. A similar tendency was seen among patients with less weight loss at nadir. The effect assumed a U-shaped association, suggesting that a stable weight or only minor recurrent weight gain over time may be the desired long-term outcome after RYGB surgery.

MBS reduces the risk for cardiovascular events and death with an effect that is driven by weight loss to a large extent^20,21^. The expected weight trajectory for most patients includes a small to moderate degree of recurrent weight gain after the initial rapid weight loss and its nadir somewhere between 12 and 24 months after surgery^5^. However, up to 20–25% of patients may have recurrent weight gain that can be defined as a suboptimal weight-loss response. Recurrent weight gain has been reported to have negative effects on metabolic biomarkers and be associated with an increased risk for relapse of metabolic co-morbidities^22,23^.

Much focus has been diverted to achieving higher postoperative weight loss, and avoiding and treating recurrent weight gain^24^. However, as supported by the results from this study and other recent studies, a mild to modest recurrent weight gain does not appear to result in any major differences in cardiovascular and death risks^25,26^. Despite a modest recurrent weight gain, a wide majority of patients still experience a substantial weight loss at 5 years follow-up^27^. Patients with cardiovascular disease and obesity have been reported to lose less weight than those without co-morbidities, but this group still has a reduction in new incident cardiovascular events after MBS^28,29^. In addition, a small group of patients have been reported to follow a different postoperative weight trajectory represented by a continued loss of weight after the expected nadir^5,30^. Although not all patients with continued weight loss suffer from malnutrition, these patients are at risk of malnutrition, sarcopenia, and other clinical adverse events recognized as risk factors for cardiovascular events^31–33^.

Although marked recurrent weight gain remains an important trajectory to study and actively treat, there is also a need to recognize continued weight loss after reaching a nadir as a non-desirable clinical trajectory. Future research should explore tailored lifestyle, obesity management medications (OMMs), or surgical interventions to improve outcomes in patients with these non-desirable postoperative weight trajectories^34^. It is important to recognize that longer-term follow-up reaching beyond the initial postoperative period is imperative to identify patients with non-desirable trajectories to allow evaluation of the underlying cause and provide adequate interventions.

It is essential to recognize that data were analysed on a group level. Subgroups of patients who continue to lose weight may benefit further from a slight continuous weight loss. Patients with T2D have previously been described to experience slightly lower weight loss compared to patients without T2D^35^. In a subgroup multivariable analysis, T2D had a less significant association with death and MACE for patients with continuous weight loss compared to the whole study group. In addition, patients with higher BMI had a stronger association with mortality and MACE among patients with continuous weight loss, supporting the hypothesis that the higher risk among patients with continuous weight loss can be at least partly explained by loss of lean body mass (*Tables S11–S12*). Further studies are needed to evaluate biochemical differences in larger populations of patients who continue to lose weight. Octreotide may be an effective treatment for patients with a marked increase in peptide YY (PYY), but whether this treatment will be of benefit for all patients in this group or only a subgroup remains unknown^36,37^. The study was not designed to evaluate fluctuations in body weight; such variations may be associated with increased risk for adverse cardiovascular outcomes for patients with coronary artery disease and should be a focus of further studies, also after MBS^38^. Additional clinical and scientific efforts should also focus on individual behavioural factors related to eating habits, weight loss practices, substance use, and physical activity, with the aim of addressing problematic eating patterns and sedentary lifestyles^39,40^.

There are major limitations in this study. The major limitation is the high number of missing data for weight at the 5-year follow-up. After the exclusion of patients lost to follow-up for weight or other exclusion criteria, only 47.3% of the population was included. Loss to follow-up and missing data are well-recognized problems for long-term studies in bariatric surgery^41^. Patients lost to follow-up were younger and less often had hypertension at baseline but otherwise had similar characteristics to those in follow-up (*Table S4*). After multiple imputations of missing data, the negative effects of continuous weight loss remained (*Tables S5–S7*). Using nationwide mandatory registries covering death and specialized healthcare, follow-up for the study’s endpoints remained very high. The nationwide coverage and the high validity of the data both mitigate this major limitation in addition to universally standardized technique used for RYGB. Second, the study is limited by the inability to determine the causes of weight changes. There are no registrations of biomarkers, such as PYY or glucagon-like peptide 1 (GLP-1), or behavioural, nutritional, or physical evaluations. Further, non-voluntary weight loss may be part of an advanced cancer disease. However, when evaluating causes of death, the relative proportion of cancer-related deaths was not different for patients who continued to lose weight. Further, a sensitivity analysis after excluding all patients who were diagnosed with cancer within 6 years after surgery did not alter the conclusion (*Table S8–S9*). The same tendency was also seen for MACE and was consistent across all initial weight-loss groups, supporting the validity of a U-shaped weight-related risk for mortality and MACE. The third limitation is that this registry study was conducted in Sweden within a universal, publicly funded healthcare system, including a population of mainly Caucasian origin, limiting the generalizability to other ethnic populations or healthcare systems. Finally, modern OMMS were not included in this study. GLP-1 receptor agonists (GLP-1RAs) have been shown to reduce the incidence of cardiovascular events^42^ and can be used as additional treatment for recurrent weight gain following MBS^34,43^. Although the use of GLP-1RAs might lead to an underestimation of risk among patients experiencing marked recurrent weight gain, OMMs were not subsidized in Sweden for obesity management during the study period and were therefore not widely used after MBS. Only 4% of patients in this cohort received GLP-1RAs at any time during the study period, suggesting a negligible effect on the outcomes.

In conclusion, continued weight loss and marked recurrent weight gain after the initial 1–2 postoperative years were associated with a higher risk for MACE and death after RYGB. Both should be considered non-desirable trajectories requiring clinical evaluation and increased support, whereas weight stability or a small recurrent weight gain may be considered a desirable trajectory after RYGB.

## Supplementary Material

znaf170_Supplementary_Data

## Data Availability

Data cannot be shared publicly because of patient confidentiality under current Swedish legislation. Data are available from the Scandinavian Obesity Surgery Registry (contact via soreg@regionorebrolan.se), the Swedish Board of Health and Welfare (contact via Registerservice@socialstyrelsen.se), and Statistics Sweden (contact via mikrodata@scb.se) for researchers who meet the criteria for access to confidential data.
