# Supplementary material for: The effect of weight change on death and cardiovascular events after Roux-en-Y gastric bypass
Source: Br J Surg. 2025 Aug 9;112(8):znaf170. doi: 10.1093/bjs/znaf170 (PMC12342772; doi:10.1093/bjs/znaf170)
Supplement: znaf170_Supplementary_Data [file znaf170_supplementary_data.docx]

**The effect of weight change on mortality and cardiovascular events after Roux-en-Y gastric bypass.**

Erik Stenberg MD, PhD^1^; Erik Näslund MD, PhD^2^; Yang Cao PhD^3,4^; Johan Ottosson MD, PhD^1^; Ingmar Näslund MD, PhD^1^

^1^Department of Surgery, Faculty of Medicine and Health, Örebro University, Örebro, Sweden

^2^Division of Surgery, Department of Clinical Sciences, Danderyd Hospital, Karolinska Institutet, Stockholm, Sweden

^3^Clinical Epidemiology and Biostatistics, School of Medical Sciences, Örebro University, Örebro, Sweden

^4^Unit of Integrative Epidemiology, Institute of Environmental Medicine, Karolinska Institutet, Stockholm, Sweden

**Corresponding author.** Erik Stenberg, Department of Surgery, Örebro University Hospital, 70182 Örebro, Sweden

**ORCID ID** [0000-0001-9189-0093](javascript:popup_orcidDetail('https://orcid.org'%20,'0000-0001-9189-0093');)

**Twitter** ErikStenberg_MD

**Supplementary Materials - Index**

| **Supplementary Tables** |  |
| --- | --- |
| Table S1. Baseline characteristics of the study group stratified by weight change from nadir weight loss after Roux-en-Y gastric bypass | *page 3* |
| Table S2. Cause of death | *page 4* |
| Table S3. Cause of death stratified by nadir weight loss | *page 5* |
| Table S4. Baseline characteristics of the study group | *page 6* |
| Table S5. Baseline characteristics of the sensitivity study group after multiple imputations | *page 7* |
| Table S6. Incidence rate and risk for mortality based on nadir total weight loss and weight change from nadir after multiple imputations | *page 8* |
| Table S7. Incidence rate and risk for major adverse cardiovascular events based on nadir total weight loss and weight change from nadir after multiple imputations | *page 9* |
| Table S8. Incidence rate and risk for mortality based on nadir total weight loss and weight change from nadir excluding patients diagnosed with cancer up until 6 years after surgery | *page 10* |
| Table S9. Incidence rate and risk for major adverse cardiovascular events based on nadir total weight loss and weight change from nadir excluding patients diagnosed with cancer up until 6 years after surgery | *page 11* |
| Table S10. Cause of death among individuals excluded due to mortality within 5 years after surgery | *page 12* |
| Table S11. Multivariable model assessing risk for mortality among patients with continuous weight loss after the expected nadir compared to all patients in the study cohort | *page 13* |
| Table S12. Multivariable model assessing risk for major adverse cardiovascular events among patients with continuous weight loss after the expected nadir compared to all patients in the study cohort | *page 14* |

**Supplementary Appendixes**

| **Table S1.** Baseline characteristics of the study group stratified by weight change from nadir weight loss after Roux-en-Y gastric bypass | | | | |
| --- | --- | --- | --- | --- |
| Weight change from nadir^1^ | <0 | 0-20 | 20-50 | >50 |
| N, n (%) | 4377 (17.3%) | 11704 (46.4%) | 7791 (30.9%) | 1358 (5.4%) |
| Age, years, mean ± SD | 42.5 ± 11.92 | 42.4 ± 11.05 | 42.5 ± 10.96 | 40.8 ± 11.20 |
| Sex, n (%) |  |  |  |  |
| Men | 1029 (23.5%) | 2595 (22.2%) | 1872 (24.0%) | 314 (23.1%) |
| Women | 3348 (76.5%) | 9109 (77.8%) | 5919 (76.0%) | 1044 (76.9%) |
| Body Mass Index, kg/m^2^, mean ± SD | 42.9 ± 5.58 | 41.9 ± 5.04 | 41.9 ± 5.12 | 42.4 ± 5.33 |
| Comorbidity, n (%) |  |  |  |  |
| Cardiovascular comorbidity | 260 (5.9%) | 538 (4.6%) | 403 (5.2%) | 65 (4.8%) |
| Sleep apnea | 510 (11.7%) | 1182 (10.1%) | 830 (10.7%) | 155 (11.4%) |
| Hypertension | 1351 (30.9%) | 3142 (26.8%) | 2288 (29.4%) | 353 (26.0%) |
| Type-2 diabetes | 812 (18.6%) | 1573 (13.4%) | 1278 (16.4%) | 224 (16.5%) |
| Dyslipidemia | 542 (12.4%) | 1187 (10.1%) | 893 (11.5%) | 144 (10.6%) |
| Depression | 720 (16.4%) | 1403 (12.0%) | 1053 (13.5%) | 295 (21.7%) |
| Smoking status, n (%) |  |  |  |  |
| Yes | 1334 (30.5%) | 3126 (26.7%) | 1862 (23.9%) | 328 (24.2%) |
| No | 2728 (62.3%) | 7794 (66.6%) | 5446 (69.9%) | 938 (69.1%) |
| Unknown | 315 (7.2%) | 784 (6.7%) | 483 (6.2%) | 92 (6.8%) |
| Level of Education, n (%) |  |  |  |  |
| Low | 802 (18.3%) | 1768 (15.1%) | 1229 (15.8%) | 241 (17.7%) |
| Mid | 2653 (60.6%) | 7192 (61.4%) | 4708 (60.4%) | 806 (59.4%) |
| High | 902 (20.6%) | 2698 (23.1%) | 1822 (23.4%) | 298 (21.9%) |
| Unknown | 20 (0.5%) | 46 (0.4%) | 32 (0.4%) | 13 (1.0%) |
| Income, n (%) |  |  |  |  |
| Q1 | 1308 (29.9%) | 2748 (23.5%) | 2033 (26.1%) | 460 (33.9%) |
| Q2 | 1125 (25.7%) | 2919 (24.9%) | 1946 (25.0%) | 343 (25.3%) |
| Q3 | 1138 (26.0%) | 3327 (28.4%) | 2115 (27.1%) | 355 (26.1%) |
| Q4 | 802 (18.3%) | 2707 (23.1%) | 1696 (21.8%) | 200 (18.3%) |
| Unknown | 3 (0.1%) | 3 (0.0%) | 1 (0.0%) | 0 (0.0%) |

N = numbers; SD = Standard deviation Q = quartile

1. Weight change in percentage of weight lost from surgery until 5-year follow-up

| **Table S2**. Cause of death | | | | |  |  |  |  |
| --- | --- | --- | --- | --- | --- | --- | --- | --- |
| Weight change from nadir | Cardiovascular | Cancer | Infection | Suicide, intoxication or accident | Respiratory disorder | Liver disease or alcohol | Kidney disease | Other cause |
| <0%^1^ | 66 (32.0%) | 54 (26.2%) | 11 (5.3%) | 31 (15.7%) | 14 (6.8%) | 9 (4.4%) | 2 (1.5%) | 18 (8.7%) |
| 0-20%^1^ | 75 (29.0%) | 61 (23.6%) | 12 (4.6%) | 60 (23.2%) | 6 (2.3%) | 15 (5.8%) | 2 (0.8%) | 28 (10.8%) |
| 20-50%^1^ | 62 (31.2%) | 48 (24.1%) | 12 (6.0%) | 43 (21.6%) | 4 (2.0%) | 17 (8.5%) | 2 (1.0%) | 11 (5.5%) |
| >50%^1^ | 5 (11.6%) | 12 (27.9%) | 6 (14.0%) | 10 (23.3%) | 2 (4.7%) | 4 (9.3%) | 0 (0.0%) | 4 (9.3%) |

1. Weight change in percentage of weight lost from surgery until 5-year follow-up

| **Table S3**. Cause of death stratified by nadir weight loss | | | | | | | | |
| --- | --- | --- | --- | --- | --- | --- | --- | --- |
| Nadir total weight loss <20% | | | | |  |  |  |  |
| Weight change from nadir^1^ | Cardiovascular | Cancer | Infection | Suicide, intoxication or accident | Respiratory disorder | Liver disease or alcohol | Kidney disease | Other cause |
| <0% | 13 (46.4%) | 3 (10.7%) | 2 (7.1%) | 3 (10.7%) | 1 (3.6%) | 2 (7.1%) | 1 (3.6%) | 3 (10.7%) |
| 0-20% | 7 (43.8%) | 6 (37.5%) | 0 (0.0%) | 2 (12.5%) | 0 (0.0%) | 1 (6.3%) | 0 (0.0%) | 0 (0.0%) |
| 20-50% | 10 (62.5%) | 2 (12.5%) | 0 (0.0%) | 2 (12.5%) | 0 (0.0%) | 1 (6.3%) | 0 (0.0%) | 1 (6.3%) |
| >50% | 1 (12.5%) | 1 (12.5%) | 2 (25.0%) | 2 (25.0%) | 0 (0.0%) | 1 (12.5%) | 0 (0.0%) | 1 (12.5%) |
| Nadir total weight loss 20 -35% | | | | |  |  |  |  |
| Weight change from nadir^1^ |  |  |  |  |  |  |  |  |
| <0% | 40 (32.5%) | 33 (26.8%) | 6 (4.9%) | 16 (13.0%) | 8 (6.5%) | 7 (5.7%) | 1 (0.8%) | 12 (9.8%) |
| 0-20% | 40 (29.4%) | 35 (25.7%) | 9 (6.6%) | 23 (16.9%) | 2 (1.5%) | 9 (6.6%) | 2 (1.5%) | 16 (11.8%) |
| 20-50% | 26 (23.2%) | 30 (26.8%) | 8 (7.1%) | 24 (21.4%) | 3 (2.7%) | 11 (9.8%) | 2 (1.8%) | 8 (7.1%) |
| >50% | 4 (14.3%) | 8 (26.8%) | 3 (10.7%) | 6 (21.4%) | 1 (3.6%) | 3 (10.7%) | 0 (0.0%) | 3 (10.7%) |
| Nadir total weight loss >35% | | | | |  |  |  |  |
| Weight change from nadir^1^ |  |  |  |  |  |  |  |  |
| <0% | 13 (23.6%) | 18 (32.7%) | 3 (5.5%) | 12 (21.8%) | 5 (9.1%) | 0 (0.0%) | 1 (1.8%) | 3 (5.5%) |
| 0-20% | 28 (26.2%) | 20 (18.7%) | 3 (2.8%) | 35 (32.7%) | 4 (3.7%) | 5 (4.7%) | 0 (0.0%) | 12 (11.2%) |
| 20-50% | 26 (36.6%) | 16 (22.5%) | 4 (5.6%) | 17 (23.9%) | 1 (1.4%) | 5 (7.0%) | 0 (0.0%) | 2 (2.8%) |
| >50% | 0 (0.0%) | 3 (42.9%) | 1 (14.3%) | 2 (28.6%) | 1 (14.3%) | 0 (0.0%) | 0 (0.0%) | 0 (0.0%) |

1. Weight change in percentage of weight lost from surgery until 5-year follow-up

| **Table S4.** Baseline characteristics of the study group | | | |
| --- | --- | --- | --- |
|  | Study group | Not included | SMD |
| N | 25230 | 26750 |  |
| Age, years, mean ± SD | 42.5 ± 11.19 | 39.4 ± 10.96 | 0.280 |
| Sex, n (%) |  |  |  |
| Men | 5810 (23.0%) | 6812 (25.5%) | 0.058 |
| Women | 19420 (77.0%) | 19939 (74.5%) | 0.058 |
| Body Mass Index, kg/m^2^, mean ± SD | 42.1 ± 5.19 | 42.4 ± 5.63 | 0.055 |
| Comorbidity |  |  |  |
| Cardiovascular comorbidity, n (%) | 1266 (5.0%) | 1089 (4.1%) | 0.043 |
| Sleep apnea, n (%) | 2677 (10.6%) | 2510 (9.4%) | 0.040 |
| Hypertension, n (%) | 7134 (28.3%) | 6061 (22.7%) | 0.129 |
| Type-2 diabetes, n (%) | 3887 (15.4%) | 3553 (13.3%) | 0.060 |
| Dyslipidemia, n (%) | 2766 (11.0%) | 2335 (8.7%) | 0.077 |
| Depression, n (%) | 3471 (13.8%) | 4230 (15.8%) | 0.056 |
| Smoking status, n (%) |  |  |  |
| Active or history of smoking | 6650 (26.4%) | 6848 (25.6%) | 0.018 |
| Level of Education, n (%) |  |  |  |
| Low | 4040 (16.0%) | 4953 (18.6%) | 0.069 |
| Mid | 15359 (60.9%) | 15617 (58.8%) | 0.043 |
| High | 5720 (22.7%) | 5992 (22.6%) | 0.002 |
| Unknown | 111 (0.4%) | 188 (0.7%) | 0.041 |
| Income, n (%) |  |  |  |
| Q1 | 6549 (26.0%) | 7890 (29.5%) | 0.078 |
| Q2 | 6333 (25.1%) | 6673 (25.0%) | 0.002 |
| Q3 | 6936 (27.5%) | 6744 (25.2%) | 0.052 |
| Q4 | 5405 (21.4%) | 5407 (20.2%) | 0.030 |
| Unknown | 7 (0.0%) | 36 (0.1%) | 0.045 |

N = number; SD = Standard deviation; Q = Quartile; SMD = Standardized mean difference

| **Table S5.** Baseline characteristics of the sensitivity study group after multiple imputations | |
| --- | --- |
|  | Sensitivity group |
| N | 51980 |
| Age, years, mean ± SD | 40.89 ± 11.18 |
| Sex, n (%) |  |
| Men | 12622 (24.3%) |
| Women | 39358 (75.7%) |
| Body Mass Index, kg/m^2^, mean ± SD | 42.26 ± 5.42 |
| Comorbidity |  |
| Cardiovascular comorbidity, n (%) | 2355 (4.5%) |
| Sleep apnea, n (%) | 5187 (10.0%) |
| Hypertension, n (%) | 13195 (25.4%) |
| Type-2 diabetes, n (%) | 7440 (14.3%) |
| Dyslipidemia, n (%) | 5101 (9.8%) |
| Depression, n (%) | 7701 (14.8%) |
| Smoking status, n (%) |  |
| Active or history of smoking | 13498 (26.0%) |
| Level of Education, n (%) |  |
| Low | 9048 (17.4%) |
| Mid | 31161 (60.0%) |
| High | 11771 (22.6%) |
| Income, n (%) |  |
| Q1 | 14454 (27.8%) |
| Q2 | 13012 (25.0%) |
| Q3 | 13691 (26.4%) |
| Q4 | 10823 (20.8%) |

N = number; SD = Standard deviation; Q = Quartile

| **Table S6**. Incidence rate and risk for mortality based on nadir total weight loss and weight change from nadir after multiple imputations | | | | |
| --- | --- | --- | --- | --- |
| Initial total weight loss <20% | | | | |
| Weight change from nadir^1^ | Incidence rate / 1000 person-years | HR (95%CI) | Adjusted HR (95%CI) | P* |
| <0% | 5.96 (4.55-7.80) | 1.00 (0.65-1.54) | 0.90 (0.58-1.39) | 0.628 |
| 0-20% | 5.98 (4.30-8.34) | Ref | Ref | Ref |
| 20-50% | 5.14 (3.74-7.06) | 0.85 (0.54-1.35) | 0.77 (0.49-1.23) | 0.279 |
| >50% | 4.70 (3.12-7.80) | 0.78 (0.46-1.32) | 0.87 (0.51-1.49) | 0.616 |
| Initial total weight loss 20 -35% | | | | |
| Weight change from nadir^1^ | Incidence rate / 1000 person-years | HR (95%CI) | Adjusted HR (95%CI) | P* |
| <0% | 3.92 (3.48-4.41) | 1.31 (1.11-1.54) | 1.32 (1.12-1.56) | 0.001 |
| 0-20% | 2.95 (2.64-3.30) | Ref | Ref | Ref |
| 20-50% | 2.98 (2.66-3.53) | 1.01 (0.86-1.19) | 1.04 (0.89-1.27) | 0.609 |
| >50% | 3.45 (2.70-4.40) | 1.16 (0.89-1.51) | 1.29 (0.99-1.69) | 0.062 |
| Initial total weight loss >35% | | | | |
| Weight change from nadir^1^ | Incidence rate / 1000 person-years | HR (95%CI) | Adjusted HR (95%CI) | P* |
| <0% | 3.20 (2.67-3.83) | 1.64 (1.32-2.05) | 1.52 (1.22-1.90) | <0.001 |
| 0-20% | 1.93 (1.69-2.20) | Ref | Ref | Ref |
| 20-50% | 2.29 (1.96-2.68) | 1.21 (0.98-1.48) | 1.21 (0.99-1.48) | 0.073 |
| >50% | 2.71 (1.57-4.66) | 1.41 (0.80-2.46) | 1.35 (0.77-2.37) | 0.290 |

*-Adjusted for age, sex, preoperative BMI, cardiovascular disease, sleep apnea, hypertension, diabetes, dyslipidemia,

depression, income, education, and smoking

1. Weight change in percentage of weight lost from surgery until 5-year follow-up

| **Table S7**. Incidence rate and risk for major adverse cardiovascular events based on nadir total weight loss and weight change from nadir after multiple imputations | | | | |
| --- | --- | --- | --- | --- |
| Initial total weight loss <20% | | | | |
| Weight change from nadir^1^ | Incidence rate / 1000 person-years | HR (95%CI) | Adjusted HR (95%CI) | P* |
| <0% | 9.51 (7.67-11.80) | 0.91 (0.65-1.27) | 0.87 (0.62-1.21) | 0.401 |
| 0-20% | 10.49 (8.14-13.51) | Ref | Ref |  |
| 20-50%2 | 9.00 (7.05-11.47) | 0.85 (0.60-1.21) | 0.81 (0.57-1.16) | 0.254 |
| >50%3 | 8.56 (6.30-11.62) | 0.81 (0.54-1.20) | 0.96 (0.64-1.44) | 0.857 |
| Initial total weight loss 20 -35% | | | | |
| Weight change from nadir^1^ | Incidence rate / 1000 person-years | HR (95%CI) | Adjusted HR (95%CI) | P* |
| <0% | 6.91 (6.31-7.56) | 1.31 (1.16-1.49) | 1.36 (1.20-1.54) | <0.001 |
| 0-20% | 5.23 (4.81-5.70) | Ref | Ref | Ref |
| 20-50% | 5.10 (4.66-5.58) | 0.98 (0.86-1.11) | 1.00 (0.89-1.14) | 0.947 |
| >50% | 5.64 (4.66-6.83) | 1.07 (0.87-1.32) | 1.22 (0.99-1.50) | 0.065 |
| Initial total weight loss >35% | | | | |
| Weight change from nadir^1^ | Incidence rate / 1000 person-years | HR (95%CI) | Adjusted HR (95%CI) | P* |
| <0% | 4.72 (4.07-5.47) | 1.41 (1.18-1.69) | 1.27 (1.06-1.53) | 0.008 |
| 0-20% | 3.33 (3.01-3.68) | Ref | Ref | Ref |
| 20-50% | 3.80 (3.37-4.30) | 1.15 (0.98-1.35) | 1.15 (0.99-1.35) | 0.075 |
| >50% | 3.77 (2.37-5.98) | 1.14 (0.71-1.82) | 1.11 (0.69-1.78) | 0.673 |

*-Adjusted for age, sex, preoperative BMI, cardiovascular disease, sleep apnea, hypertension, diabetes, dyslipidemia,

depression, income, education, and smoking

1. Weight change in percentage of weight lost from surgery until 5-year follow-up

| **Table S8**. Incidence rate and risk for mortality based on nadir total weight loss and weight change from nadir excluding patients diagnosed with cancer up until 6 years after surgery | | | | |
| --- | --- | --- | --- | --- |
| Initial total weight loss <20% | | | | |
| Weight change from nadir^1^ | Incidence rate / 1000 person-years | HR (95%CI) | Adjusted HR (95%CI) | P* |
| <0% | 6.67 (4.54-9.79) | 1.74 (0.89-3.38) | 1.83 (0.93-3.61) | 0.079 |
| 0-20% | 3.89 (2.26-6.71) | Ref | Ref | Ref |
| 20-50% | 4.22 (2.55-7.01) | 1.06 (0.50-2.22) | 0.89 (0.41-1.91) | 0.763 |
| >50% | 3.63 (1.81-7.25) | 0.90 (0.37-2.18) | 1.05 (0.42-2.59) | 0.920 |
| Initial total weight loss 20 -35% | | | | |
| Weight change from nadir^1^ | Incidence rate / 1000 person-years | HR (95%CI) | Adjusted HR (95%CI) | P* |
| <0% | 3.62 (2.97-4.41) | 1.53 (1.17-1.99) | 1.56 (1.19-2.03) | 0.001 |
| 0-20% | 2.29 (1.92-2.73) | Ref | Ref | Ref |
| 20-50% | 2.20 (1.81-2.68) | 0.97 (0.75-1.26) | 1.00 (0.76-1.30) | 0.973 |
| >50% | 2.78 (1.88-4.11) | 1.23 (0.80-1.89) | 1.54 (1.00-2.38) | 0.050 |
| Initial total weight loss >35% | | | | |
| Weight change from nadir^1^ | Incidence rate / 1000 person-years | HR (95%CI) | Adjusted HR (95%CI) | P* |
| <0% | 3.10 (2.30-4.18) | 1.88 (1.31-2.69) | 1.67 (1.16-2.40) | 0.006 |
| 0-20% | 1.58 (1.30-1.93) | Ref | Ref | Ref |
| 20-50% | 2.15 (1.69-2.74) | 1.38 (1.01-1.89) | 1.28 (0.93-1.75) | 0.127 |
| >50% | 2.47 (1.18-5.18) | 1.62 (0.75-3.49) | 1.50 (0.70-3.24) | 0.299 |

*-Adjusted for age, sex, preoperative BMI, cardiovascular disease, sleep apnea, hypertension, diabetes, dyslipidemia, depression, income, education, and smoking

1. Weight change in percentage of weight lost from surgery until 5-year follow-up

| **Table S9**. Incidence rate and risk for major adverse cardiovascular events based on nadir total weight loss and weight change from nadir excluding patients diagnosed with cancer up until 6 years after surgery | | | | |
| --- | --- | --- | --- | --- |
| Initial total weight loss <20% | | | | |
| Weight change from nadir^1^ | Incidence rate / 1000 person-years | HR (95%CI) | Adjusted HR (95%CI) | P* |
| <0%4 | 9.70 (7.03-13.39) | 1.02 (0.63-1.65) | 1.13 (0.70-1.84) | 0.609 |
| 0-20% | 9.52 (6.70-13.54) | Ref | Ref |  |
| 20-50%2 | 8.97 (6.31-12.76) | 0.93 (0.56-1.52) | 0.88 (0.53-1.47) | 0.630 |
| >50%3 | 5.96 (3.46-10.26) | 0.61 (0.32-1.17) | 0.72 (0.37-1.39) | 0.324 |
| Initial total weight loss 20 -35% | | | | |
| Weight change from nadir^1^ | Incidence rate / 1000 person-years | HR (95%CI) | Adjusted HR (95%CI) | P* |
| <0% | 6.73 (5.81-7.79) | 1.38 (1.14-1.66) | 1.45 (1.19-1.75) | <0.001 |
| 0-20% | 4.81 (4.23-5.44) | Ref | Ref | Ref |
| 20-50% | 4.43 (3.86-5.09) | 0.93 (0.77-1.12) | 0.96 (0.80-1.16) | 0.689 |
| >50% | 5.17 (3.87-6.90) | 1.08 (0.79-1.48) | 1.44 (1.05-1.97) | 0.025 |
| Initial total weight loss >35% | | | | |
| Weight change from nadir^1^ | Incidence rate / 1000 person-years | HR (95%CI) | Adjusted HR (95%CI) | P* |
| <0% | 4.96 (3.91-6.29) | 1.66 (1.25-2.19) | 1.38 (1.04-1.83) | 0.025 |
| 0-20% | 2.93 (2.53-3.39) | Ref | Ref | Ref |
| 20-50% | 3.78 (3.14-4.54) | 1.31 (1.03-1.65) | 1.19 (0.94-1.51) | 0.146 |
| >50% | 3.18 (1.65-6.10) | 1.11 (0.57-2.16) | 1.00 (0.51-1.96) | 0.997 |

*-Adjusted for age, sex, preoperative BMI, cardiovascular disease, sleep apnea, hypertension, diabetes, dyslipidemia,

depression, income, education and smoking

1. Weight change in percentage of weight lost from surgery until 5-year follow-up

| **Table S10**. Cause of death among individuals excluded due to mortality within 5 years after surgery | | | | | | | | |
| --- | --- | --- | --- | --- | --- | --- | --- | --- |
|  | Cardiovascular | Cancer | Infection | Suicide, intoxication or accident | Respiratory disorder | Liver disease or alcohol | Kidney disease | Other cause |
| N (%) | 158 (22.6%) | 150 (21.5%) | 33 (4.7%) | 249 (35.6%) | 17 (2.4%) | 29 (4.1%) | 7 (1.0%) | 56 (8.0%) |

| **Table S11.** Multivariable model assessing risk for mortality among patients with continuous weight loss after the expected nadir compared to all patients in the study cohort | | | | |
| --- | --- | --- | --- | --- |
|  | Continuous weight loss^1^ | P^1^ | Study cohort^1^ | P^1^ |
| Sex, male | 2.17 (1.61-2.92) | <0.001 | 2.04 (1.74-2.40) | <0.001 |
| Men |  |  |  |  |
| Age, years | 1.08 (1.07-1.10) | <0.001 | 1.07 (1.06-1.07) | <0.001 |
| Body Mass Index, BMI unit | 1.02 (1.00-1.05) | 0.049 | 1.01 (1.00-1.02) | 0.182 |
| Cardiovascular comorbidity | 1.27 (0.87-1.87) | 0.216 | 1.34 (1.08-1.66) | 0.008 |
| Sleep apnea | 0.74 (0.51-1.07) | 0.109 | 1.04 (0.85-1.26) | 0.718 |
| Hypertension | 1.62 (1.15-2.27) | 0.005 | 1.30 (1.09-1.55) | 0.004 |
| Type-2 diabetes | 0.96 (0.69-1.34) | 0.809 | 1.33 (1.12-1.59) | 0.001 |
| Dyslipidemia | 1.06 (0.74-1.51) | 0.746 | 1.06 (0.86-1.29) | 0.544 |
| Depression | 0.87 (0.58-1.30) | 0.493 | 1.16 (0.94-1.43) | 0.165 |
| Active or history of smoking | 1.55 (1.14-2.10) | 0.005 | 1.59 (1.35-1.87) | <0.001 |
| Level of Education |  |  |  |  |
| Low | Reference | Reference | Reference | Reference |
| Mid | 0.90 (0.64-1.26) | 0.533 | 0.95 (0.79-1.14) | 0.533 |
| High | 0.97 (0.63-1.50) | 0.897 | 0.85 (0.67-1.08) | 0.897 |
| Income |  |  |  |  |
| Q1 | Reference | Reference | Reference | Reference |
| Q2 | 0.71 (0.49-1.02) | 0.062 | 0.68 (0.56-0.82) | <0.001 |
| Q3 | 0.86 (0.60-1.23) | 0.415 | 0.61 (0.50-0.74) | <0.001 |
| Q4 | 0.65 (0.42-0.99) | 0.046 | 0.51 (0.40-0.65) | <0.001 |

BMI = Body Mass Index; Q = Quartile

1 – Multivariable Cox regression model including all variables in the Table.

| **Table S12.** Multivariable model assessing risk for major adverse cardiovascular events among patients with continuous weight loss after the expected nadir compared to all patients in the study cohort | | | | |
| --- | --- | --- | --- | --- |
|  | Continuous weight loss^1^ | P^1^ | Study cohort^1^ | P^1^ |
| Sex, male | 1.75 (1.38-2.22) | <0.001 | 1.79 (1.58-2.02) | <0.001 |
| Age, years | 1.08 (1.06-1.09) | <0.001 | 1.06 (1.06-1.07) | <0.001 |
| Body Mass Index, BMI unit | 1.03 (1.01-1.05) | 0.010 | 1.00 (0.99-1.01) | 0.552 |
| Cardiovascular comorbidity | 1.61 (1.20-2.15) | 0.001 | 1.85 (1-59-2.15) | <0.001 |
| Sleep apnea | 1.00 (0.76-1.32) | 0.998 | 1.03 (0.89-1.19) | 0.661 |
| Hypertension | 1.64 (1.25-2.14) | <0.001 | 1.49 (1.30-1.70) | <0.001 |
| Type-2 diabetes | 1.21 (0.94-1.56) | 0.136 | 1.42 (1.25-1.62) | <0.001 |
| Dyslipidemia | 0.97 (0.74-1.27) | 0.814 | 1.12 (0.97-1.28) | 0.129 |
| Depression | 1.09 (0.81-1.46) | 0.586 | 1.19 (1.02-1.39) | 0.025 |
| Active or history of smoking | 1.35 (1.06-1.72) | 0.015 | 1.39 (1.23-1.57) | <0.001 |
| Level of Education |  |  |  |  |
| Low | Reference | Reference | Reference | Reference |
| Mid | 0.86 (0.66-1.12) | 0.269 | 0.88 (0.77-1.01) | 0.072 |
| High | 0.77 (0.54-1.08) | 0.133 | 0.78 (0.65-0.93) | 0.006 |
| Income |  |  |  |  |
| Q1 | Reference | Reference | Reference | Reference |
| Q2 | 0.71 (0.54-0.95) | 0.020 | 0.76 (0.66-0.88) | <0.001 |
| Q3 | 0.88 (0.66-1.16) | 0.354 | 0.78 (0.65-0.88) | <0.001 |
| Q4 | 0.72 (0.51-1.00) | 0.052 | 0.64 (0.53-0.76) | <0.001 |

BMI = Body Mass Index; Q = Quartile

1 – Multivariable Cox regression model including all variables in the Table.
